# Influence of Coupling Agent in Mechanical, Physical and Thermal Properties of Polypropylene/Bamboo Fiber Composites: Under Natural Outdoor Aging

**DOI:** 10.3390/polym12040929

**Published:** 2020-04-17

**Authors:** Lety del Pilar Fajardo Cabrera de Lima, Ruth Marlene Campomanes Santana, Cristian David Chamorro Rodríguez

**Affiliations:** 1 University of Valle, Cali 13 #100-00, Colombia; cristian.chamorro@correounivalle.edu.co; 2 Metallurgical and Materials Engineering, Federal University of Rio Grande do Sul, Porto Alegre 9500-970, Brazil; ruth.santana@ufrgs.br

**Keywords:** coupling agent, natural aging, bamboo fiber, waste, composites, properties

## Abstract

Researches on thermoplastic composites using natural fiber as reinforcement are increasing, but studies of durability over time are scarce. In this sense the objective of this study is to evaluate changes in the properties of polypropylene/bamboo fiber (PP/BF) composite and the influence of the use of coupling agent (CA) in these composites after natural ageing. The PP/BF (70/30 wt) composites and 3% wt CA (citric acid from natural origin and maleic anhydride grafted polypropylene from petrochemical origin) were prepared by using an internal mixer chamber and then injection-molded. The samples were exposed to natural weathering for a total period of 12 months and characterized before and after exposure. All exposed composites experienced a decrease in their properties, however, the use of CA promoted more stability; in mechanical properties, the composites with CA showed lower loss about 23% in Young′s modulus, 18% in tensile stress at break, and 6% in impact strength. This behavior was similar in thermal and physical properties, the result for the CA of natural origin being similar to that of synthetic origin. These results indicate that the use of a CA may promote higher interaction between the fiber and the polymer. In addition, the CAs of organic origin and synthetic origin exhibited similar responses to natural ageing.

## 1. Introduction

Interest in obtaining composite materials reinforced with fibers from renewable sources, specifically lignocellulosic fibers, has grown in recent years. Lignocellulosic fibers are added to polymers to obtain composites; when used as reinforcing agents they impart certain benefits such as low density, high specific strength, and high modulus [1]. This type of composites is known as wood plastic compounds (WPCs), which have a great potential in application as a new wood substitute [2]; they offer advantages in relation to insect attacks, water absorption, and degradation. These WPCs entail considerably lower maintenance expenses, have higher durability and are easy to clean and mold. Currently, the development of products made from plastic and fiber wood is increasing [3]. Its malleability allows obtaining products of varied shapes, and easy reproduction of building elements. These composites offer applications in different industries such as automotive components, construction applications, footwear, interior and street furniture, among others. This type of material can replace wood in many applications. According to ASTM D6662 [4], WPC can be defined as a manufactured product with a plastic content higher than 50% by mass and a rectangular cross section; it has dimensions typical of industrialized wood products. Advances in this field have allowed the term to be applied to wood plastic compounds of other formats and diverse applications. There is a drawback in obtaining WPCs, this is the polarity difference; the lignocellulosic fibers have hydrophilic character, which is incompatible with the hydrophobic character of the polymer matrix [5]. This polarity difference leads to a poor interfacial bonding between the fiber and matrix [6,7], that can influence weak mechanical properties of composites. To enhance the interfacial bonding between fiber and matrix, the coupling agent (CA) is added; in WPCs whose matrix is polypropylene (PP), the most commonly reported and used coupling agent is the maleic anhydride grafted polypropylene (MA), this is of petrochemical source. The purpose of this modification is the insertion of polar clusters in the main chain of the polypropylene, making it able to establish physical and/or chemical interactions with other materials [8]. Some authors have studied the use of organic acids as coupling agents, myristic acid [8], stearic acid [9], and caprylic acid [10], the results suggest that carboxylic acids are compounds from a vegetal source that present a chemical structure that favors compatibilization, so they can be used as coupling agents. In this study, two CAs were tested, a commercial MA of petrochemical source, and citric acid (CI) of vegetal source.

Among lignocellulosic fibers, there are several options for the reinforcement of composite materials. The bamboo fibers (BF) can be a renewable and cheaper substitute for synthetic fibers in some application fields [11]. Bamboo is known for its rapid renewal and growth can be used at an age of 3 to 4 years, compared to wood, bamboo can renew itself much more rapidly [11,12]. The structural properties of bamboo based on the resistance/specific mass and rigidity/specific mass ratios surpass those of wood and concrete and are even comparable to those of steel [13]. It is well known that bamboo has numerous applications in different fields, including its industrialization in the manufacture of laminates, floors, beams, and plywood panels, among others. However, the related processing generates a high amount of waste. This waste can be recycled and reused to contribute more economic and social benefits [14]. Due to the morphological characteristics of bamboo, it cannot be used in its entirety; hence, in the present study, we take advantage of bamboo residues for use as reinforcing agent in WPCs. 

Among the conventional polymers commonly used in thermoplastic composites is polypropylene (PP), characterized by its great versatility, lightness, favorable mechanical properties [2,11,12], and easyness for processing, according to the properties required for product performance. PP is increasingly used in lignocellulosic fibers reinforced polymeric composites due to its higher melt flowability when compared to other polyolefins [15]. 

Most polymeric materials are sensitive to weathering, a process in which the incidence of ultraviolet (UV) radiation and atmospheric oxygen interacts. The chemical reactions involved can be accelerated due to solar heating of the component or by the presence of tensions (applied externally or residual from the process [16,17]. This degradation is characterized as a physical-chemical process, which leads to the cleavage of polymer chains. This process depends on four factors (environmental conditions, polymer type, processing conditions and polymer structure) and is responsible for the loss of some physical properties due to fragmentation, resulting in a modification of the mechanical properties [18,19]. The use of WPC by the construction industry has resulted in concern about the durability of these products after weathering; some articles have reported the weathering often is a response for the loss of mechanical properties [20].

In polymeric materials, a series of chemical-oxidative reactions that affect the performance of the material occurs; two causes for this phenomenon are the cleavage of the chains of higher molecular weight and the formation of cracks on the surface [21,22]. The photo-degradation mechanism primarily involves the absorption of UV radiation, and subsequent oxidative reactions occur in auto-catalytic processes, causing a reduction in the molecular weight and alterations in the chemical structure. Among the factors that can promote degradation in polymeric materials are: heat, light, oxygen, UV radiation, humidity, and pollutants [23,24,25,26]. It is very important to evaluate the stability of the polymeric materials to determine the effect of the environmental conditions in the aging process. Thus, it is necessary to test the shelf-life under environmental real conditions. Natural aging is a useful evaluative process for studying the degradation of polymeric materials [27]. In general, this process is related to molecular cleavage, resulting in shorter chains. In this study, composites of PP matrix reinforced with BF were prepared in three groups of PP/FB composites: 1° without CA, 2° with CA (MA) and 3° with CA (CI). The composites were exposed under natural aging for a total period of 12 months, in order to evaluate changes in the mechanical, physical, and thermal properties of PP/BF composites under natural aging and the influence of the use of CA of petrochemical source and CA of natural source.

## 2. Materials and Methods 

### 2.1. Materials

The materials used in this study were PP with MFI 45 g/10 min (230 °C/2,16Kg), manufactured by the company Braskem (Porto Alegre, RS, Brazil); polypropylene grafted with maleic anhydride (MA), (PolyBond® 3200, Addivant-molecular weight of 42000 g/mol and acid number of 11 mg KOH/g) was provided by Clariant; citric acid, CI, manufactured by the company Neon Comercial Ltda (Suzano, SP, Brazil); and bamboo fiber (BF) from the *Guadua angustifólia* species, with particle sizes of 250 μm, from residues generated in the industrialization of bamboo (Popayán, CA, Colombia).

### 2.2. Processing Conditions

Before processing, the BF was dried in an oven at 58 °C for 24 h to remove any moisture. The materials were weighed separately using an AY-220 analytical balance and prepared at the compositions in mass percent, it is specified in Table 1.

The materials were mixed manually, then, the mixture was transferred in an internal mixer chamber HAAKE RheoDrive 7 Rheomix OS (Karlsruhe, Germany). The samples were processed at temperature of 180 °C and a rotation speed of 60 rpm for 5 min. The test specimens were obtained by injection molding, using a mini-injector Thermo Scientific HAAKE model MiniJet II (Karlsruhe, Germany), with barrel and mold temperatures of 190 and 40 °C, respectively and a pressure of 400 bar. 

### 2.3. Natural Aging

To evaluate the stability of the polymeric materials, it is necessary to submit them to tests that simulate the real conditions of weather exposure during their useful life, for which a natural aging test is used [28].

The composites were exposed to natural aging in a system composed of a wooden structure and a nylon panel with a 45° inclination, in accordance with ASTM D 1435-05 [29], in two groups (6 and 12 months). The system was located at latitude (30°05′ South), longitude (51°11′ West) and altitude (174 m) in the humid tropical climate of the city of Porto Alegre RS (INMET, 2015). The data were monitored during the exposure time [30]. 

Table 2 shows the results of the climatological variables analyzed according to data obtained from the National Institute of Meteorology (INMET) [30] and data from the Climate Weather site for the city of Porto Alegre [31]. 

In the first 6 months (09/01/2016-09/07/2016), corresponding to the summer and autumn seasons, the first group of composites faced extreme atmospheric conditions, with high temperatures, a high level of rainfall and an UV index above 11, even reaching high rates (14) classified as extreme according to the World Health Organization (WHO). During this period, the total rainfall was approximately 943 mm, and the maximum temperatures varied between 28 and 36 °C, and minimum temperatures average of 19 °C. This period was affected by the “El Niño” phenomenon, which is characterized by extreme weather events; thus, at the end of January, a downburst occurred, which, according to the integrated command center of the city of Porto Alegre (CEIC) [32], indicated an atmospheric explosion that may result in destructive events with strength similar to that of a tornado. In March, there were high average temperatures; in this case, the system causing this heat wave was the cyclical “El Niño” phenomenon. In April, a temperature of 36 °C was registered, which, according to the INMET, is the highest temperature for this month since 1916 [30]. The second group of composites was subjected to all four seasons (09/01/2016-09/01/2017); in addition to high and low temperatures, a high level of rainfall, extreme UV index values, and a high average variation can be observed. The winter (June, July, and August) was marked by rain, lower temperatures and cold waves, which decreased throughout the season. In the month of August, there were higher temperatures (33.6 °C), with historical values 15 °C above average for this time of year according to the CEIC. These trends were caused by the “El Niño” phenomenon (weakening of the phenomenon) [32]. The spring (September, October, and November) was characterized by the entry of “La Niña”, with a prolongation of low temperatures and a reduction in rainfall in this region of Brazil. The geographical location of the study offered severe weather conditions, as it is relatively close to the Equatorial line and very close to the South Pole. This region is characterized by the existence of a hole in the ozone layer as a consequence of the constant emission of polluting gases into the atmosphere and the burning of fuel from aircraft flying at heights where the layer is more concentrated. These gases are taken to the poles by air currents, and once they make contact with ozone, they react and destroy the ozone layer [33].

## 3. Characterization

### 3.1. Mechanical Tests

For mechanical tests, seven test specimens were used for each formulation. The specimens were evaluated before (0 months) and after (6 and 12 months) natural aging. A tensile test was carried out in an Instron model 4200 universal test machine, in accordance with ASTM D638, using a 5-kN cell load and a speed of 2 mm/min. An impact test was carried out in accordance with ASTM D256, with the IZOD impact test on a CEAST model Impactor II using a 2.75 J hammer without an entangler. Mechanical tests were carried out in the laboratory of tests of polymeric materials (LAPOL) of the Federal University of Rio Grande do Sul (LAPOL/UFRGS).

### 3.2. Thermal Test

The thermal stability of the samples was evaluated before and after 12 months of exposure by thermogravimetric analysis (TGA), according to the ASTM E-1131 standard. The TGA equipment was programmed at a temperature between 25 to 1000 °C with a heating rate of 20 °C/min, under an N_2_ atmosphere. 

The melting temperatures and crystallization enthalpies of the composites were evaluated using differential scanning calorimetry (DSC) according to ASTM D3418, before and after 12 months of exposure. The samples were subjected to two stages of heating and cooling at a rate of 10 °C/min with a range of 20–200 °C. The crystallinity index of the composites was determined using the following equation:

*X*c = (Δ*H*_f_/(Δ*H*_0_(%PP)) × 100% (1)

where, *X*c is crystallinity index; Δ*H*_f_ is melting enthalpy of the sample; Δ*H*_0_ is Melting enthalpy of 100% crystalline PP, the most frequent value found in the literature was 208 J/g [34] and %PP is the percentage of PP used in the composite formulation.

### 3.3. Physical Tests

#### 3.3.1. Morphological Evaluation

The microscopy analysis was carried out with a scanning electron microscopy (SEM) machine (JEOL JSM6060) operating at 15 kV. The morphology composite surfaces were evaluated before and after natural aging, the samples were sputtered with gold before the SEM analysis, at a 400X magnification using a scanning electron microscope.

#### 3.3.2. Optical Analysis

The optical test was carried out according ASTM D2244-14, were evaluated properties of brightness and luminosity before and after 6 and 12 months of exposure. For this analysis a spectrometer, BYK-Gardnes Spector Guide model Sphere Gloss was used.

#### 3.3.3. Melt Flow Index

The melt flow index test of composites before and after 6 and 12 months of exposure natural weathering were performed on the CEAST Modular Melt Flow Model 7026.000 equipment (Karlsruhe, Germany), according to Method A of ASTM D1238. The conditions used were based on polypropylene 230 °C/2.16 kg, with residence time of 4 min.

### 3.4. Statistical Analysis

The results of the mechanical properties of the composites with and without CA, (before and after natural aging) were analyzed with the statistical tool ANOVA of single factor in commercial software for a level of reliability of 95% in the Software Statistical 12, results of the ANOVA are represented in the tables through letters. Equal letters in the same column indicated that there were no significant differences between composites.

## 4. Results and Discussion

### 4.1. Mechanical Properties

**Figure 1:**
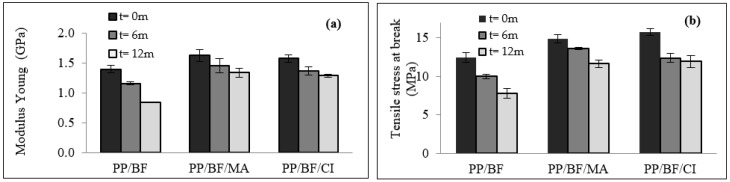
Tensile test results of composites exposed to weathering: (**a**) Modulus Young; (**b**) Tensile stress at break.

The results of the elastic modulus and tensile strength at rupture as a function of exposure time are presented in Figure 1a,b.

Figure 1a shows that all exposed composites experienced a decrease in Young’s modulus. The composites without a CA (PP/FB) presented a higher loss (39%) after 12 months of exposure to natural aging. The composites with a CA (PP/BF/MA and PP/BF/CI) showed lower loss compared with PP/BF composites of 17% and 16%, respectively. Thus, the use of a CA provided higher stability to the composites, with the result for the CA of natural origin being similar to that of synthetic origin, similar behavior was observed by Poletto et al. [10] where was studied the use of natural oils as coupling agents in wood flour and recycled polypropylene composites, they verified that the improvement influenced by caprylic acid was similar to that promoted by the addition of MA. 

In Figure 1b, the results of tension at rupture exhibit a similar behavior; all composites presented a loss according to the exposure time. The composites without CA presented a decrease of 37%, and the composites with CA presented lower loss, approximately 21%; thus, the interference of CA of natural origin (CI) was equal to that of synthetic origin (MA). The introduction of CA improved the interfacial compatibility between lignocellulosic fiber and polymer matrix [35,36].

Other authors who studied the effect of the coupling agent on bamboo fiber reinforced polypropylene composites reported similar behavior, the interfacial adhesion between the filler and the matrix is reached with the addition of coupling agent [1,2,11,12]. In contrast, in the case of composites without CA, structural defects may have occurred due to a lack of homogeneous surfaces, causing higher water absorption and thus accelerating the initiation of the degradation process. In the first six months of exposure, loss of mechanical tensile properties can be observed; during this period (summer and spring), the composites were less rigid, which may be related to the absorption of water due to the high frequency of rain, rainwater may have acted as a plasticizer. Although the samples were previously oven dried, as required by ASTM standards, there is the possibility that there were still occluded water molecules inside the composites, retained by the intermolecular forces of hydrogen, especially in the samples that were exposed during all the evaluated period. Since the formation of environmental stress cracking on the surface of the exposed composites, it facilitated the diffusion of rainwater in their inside, causing the bamboo fibers to swell.

In general, this period was characterized by extreme atmospheric conditions (taking into account the conditions listed in Table 2); thus, the composites were subjected to high rates of rainfall, high temperatures, and extreme UV index values according to the WHO. These conditions led to photo-oxidative degradation, which may have favored hydrolytic degradation of holocellulose from BF and stress cracking of composite due to high rainfall. The phenomenon of tense cracking known as stress cracking can be due to two factors that affected the specimens: mechanical stress and fluid exposure (rain) [32].

**Figure 2:**
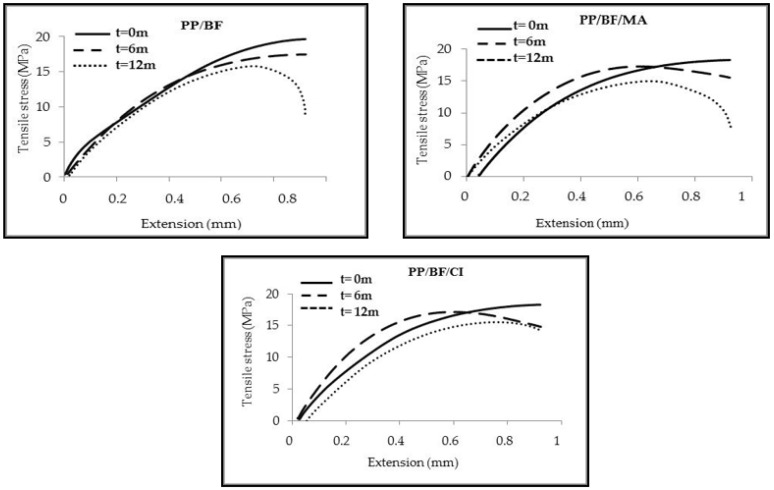
Tensile stress-extension curves of the composites before and after exposition.

**Figure 3:**
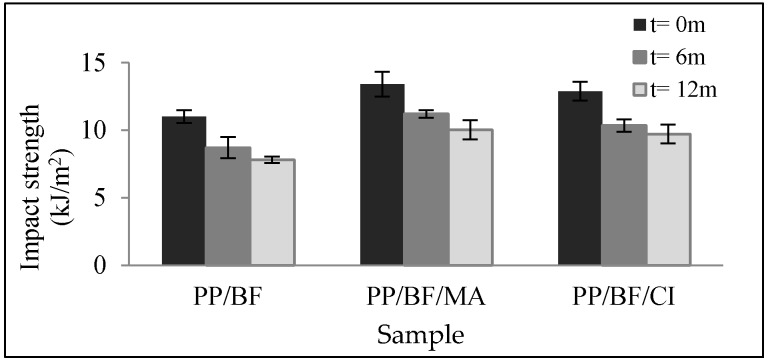
Impact strength of composites before and after natural aging

Figure 2, shows tensile stress-extension curves of the composites before and after exposition. After 12 months of exposure, all composites showed decrease in their mechanical performance, indicating higher fragility. Thus, the group of composites that remained exposed during all four seasons in addition to the climatic conditions described above, faced a winter season marked by lower average temperatures, cold snap, reduced rain and a spring season characterized by a prolongation of lower temperatures, high temperatures and less rainfall. The decrease in mechanical properties was due to several climatic factors, the most relevant factor was the high UV index [37]. The loss of mechanical tensile properties in thermoplastic composites reinforced with lignocellulosic fibers exposed to natural weathering has also been reported by other authors [38,39,40,41,42]. The decrease in Young′s modulus presented by the composites as a function of exposure time may be due to degradation caused by the cleavage of chains [38]. Figure 3 shows comparative results of impact strength of composites before and after natural ageing.

The composites with CA evaluated before natural ageing, showed significant improvement in impact strength, similar behavior was observed by other authors [1,11]. As shown in Table 3, this increase was of 22% compared to the composites without CA. In fact, the highest impact strength may be due to the presence of CA in the composite, since this allowed to attenuate the energy transfer from the matrix to the fiber. This result is corroborated by the above-mentioned results for the mechanical properties. Similar behavior was observed by Zhout et al. [11] in their study about effects of polypropylene grafted with maleic anhydride content on the physico-mechanical properties and rheological behavior of bamboo powder-polypropylene foamed composites; they verified that the impact strength increased with the addition of coupling agent, they observed a significant improvement in mechanical properties. 

After 6 and 12 months of exposure, the composites showed a similar drop, but even so, the impact strength of the composites with CA continued to be higher than the composite without CA. These values are shown in Table 3. 

The decrease in mechanical properties was higher in the first six months, as mentioned above, during this period; the exposed composites generally faced more extreme climatological variables, with the UV index considered extreme according to the WHO, resulting in photo-oxidative degradation, being more evident in the composites without CA.

Joseph et al. [38], reported a decrease in mechanical properties in PP/sisal fiber composites exposed to natural aging due to the cleavage of chains and degradation that occurs in the PP molecules as a result of photo-oxidation by UV radiation. As shown in Table 3, none statistical difference is observed between composites containing the different coupling agents (PP/BF/MA and PP/BF/CI) according with the evaluation of the statistical tool ANOVA of single factor. 

### 4.2. Thermal Properties

**Figure 4:**
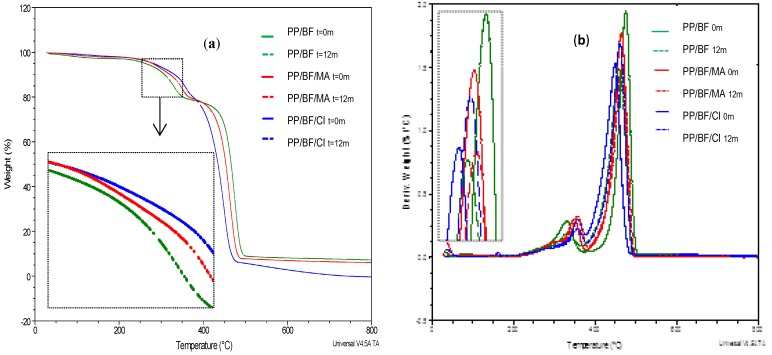
Thermal curves comparative (**a**) thermogravimetric (TGA) and (**b**) derivatives of the thermogravimetric (DTG), of the composites before and after 12 months of natural ageing.

Figure 4a,b shows the thermogravimetric curves (TGA) and the derivatives of the thermogravimetric curves (DTG) respectively, of the composites before and after 12 months of exposure.

It is observed that the composites presented four stages of mass loss, as follows: the first occurred below 100 °C and may be related to the humidity present in the fiber due to the hydrophilic characteristic, and probably due to some extractive substances with low molecular weight [43,44]; the second stage corresponds to the decomposition of the hemicellulose, in composites without CA with peak temperature of DTG at 259 °C before exposure and at 265 °C after exposure; in PP/BF/MA composites at 286 and 299 °C (before and after exposure respectively) and in PP/BF/CI composites at 295 and 289 °C (before and after exposure respectively). The third stage refers to the decomposition of the cellulose at 330 and 333 °C for the composites without CA (before and after exposure respectively), in composites PP/BF/MA at 348 and 357 °C (before and after exposure respectively) and in PP/BF/CI composites at a stable temperature of 357 °C before and after exposure. The fourth stage corresponds to the decomposition of the polymeric matrix, as shown in Table 4, decrease in the temperature peak was observed in all the composites, being a higher loss for composites without AC of 18 degrees, in composites with CA there was a lower loss of 10 and 3 degrees for PP/BF/MA and PP/BF/CI respectively. The composites with CA showed higher thermal stability than PP/FB, similar behavior was observed by others authors [45,46]. This refers to the coupled composites presented a greater interfacial interaction due to the reaction of the functional groups of the CA and the hydrophilic groups of the fiber surface [47]. This behavior may be ascribed to the reduction of motion of the matrix molecules due to presence of the modified FB with CA [11]. The PP/BF/CI composites presented higher thermal stability. According with Poletto [48], research with thermoplastic composites reinforced with wood flour, the use of organic acids promoted the thermal stability and provided a greater interfacial interaction. This result is probably due to the formation of hydrogen bonds [48] and a lower esterification reaction [48,49,50] between the hydroxyl groups of the wood flour and the carboxyl groups of the organic acid [49].

Table 4 shows results corresponding to the thermal properties of the composites, temperature data at 10% mass loss and ash percentage (TGA); peak temperature from DTG of each decomposition stage.

**Figure 5:**
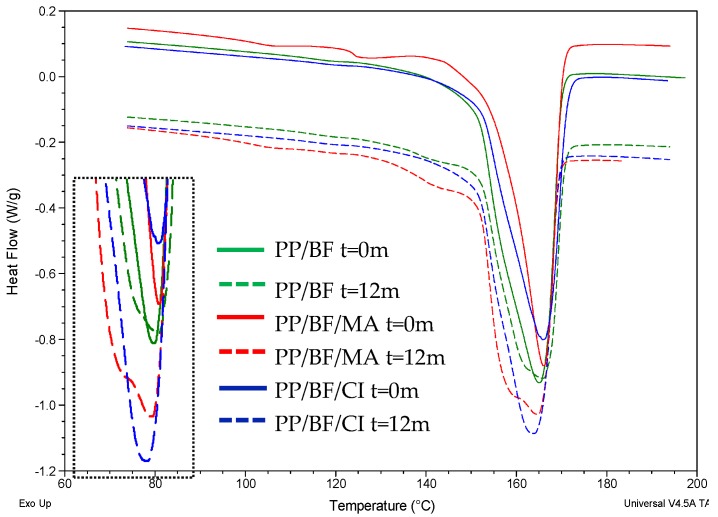
Differential scanning calorimetry (DSC) curves of the composites before and after 12 months of exposure.

Figure 5 shows the DSC comparative curves of the composites before and after 12 months of exposure. It can be observed that the composites did not show a significant change in the melting temperature; however, an increase in peak intensity was observed in the curves of the coupled composites, after exposure. In composites without CA, there was a decrease in Δ*H*_m_ after 12 months of exposure, while in composites with CA an increase after exposure was observed. The decrease of Δ*H*_m_ of PP/FB composite could be influenced by the sudden degradation by environment stress cracking, due to the poor interfacial bonding between fiber and matrix that, over 12 months of exposure to natural ageing, could have favored the diffusion of rainwater molecules in the amorphous phase of PP, this water molecules difficult to recrystallize the smaller PP chains, product of chain cleavage caused by photooxidation [51]. This was observed especially in the first six months, since this was the rainiest period with the highest temperatures, and the highest UV index.

Table 5 shows some results corresponding to the thermal properties of the compounds, by DSC, as well as the crystallinity index (*X*c) of the compounds, before and after 12 months of exposure. A decrease in the *X*c of the compounds without CA is observed, after the exposure, while in the coupled composites it showed an increase, this increase was higher in the composites with MA, this behavior is consistent with the mechanical properties, since, in composites without CA, the elastic modulus fell sharply, they became very fragile, after 12 months of exposure they presented a loss of modulus of 39%, while in composites with CA the loss of modulus values was less, 18% in PP/BF/MA and 16% for PP/BF/CI, the use of the coupling agent favors a better fiber/matrix interaction. The increase in the degree of crystallinity for the PP in composites with CA, is probably due to the fact that when the PP degrades chain cleavage of higher molecular weight occurs, and therefore are formed chains of smaller size, these that can favor the formation of crystalline phases [52].

### 4.3. Morphological Properties

**Figure 6:**
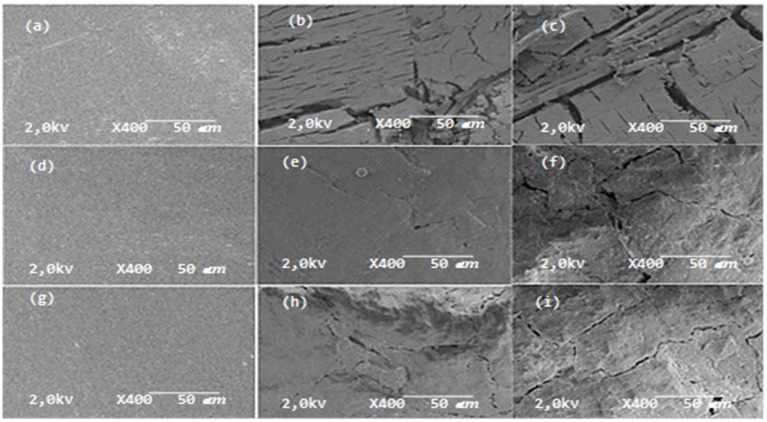
(**a**) PP/BF t = 0 m; (**b**) PP/BF t = 6 m; (**c**) PP/BF t = 12 m; (**d**) PP/BF/MA t = 0 m; (**e**) PP/BF/MA t = 6 m; (**f**) PP/BF/MA t = 12 m; (**g**) PP/BF/CI t = 0 m; (**h**) PP/BF/CI t = 6 m; (**i**) PP/BF/CI t = 12 m.

Figure 6 presents the micrographs of the composites before and after (6 and 12 months) natural aging. In all composites, the surfaces appeared altered, with the presence of cracks within the first six months of exposure. The alterations were more intense and deep with 12 months of exposure.

The composites without CA showed surfaces with deeper fractures (Figure 6b,c), while the composites with a CA showed shallower fractures (Figure 6e,f,h,i). 

These observations are consistent with the findings on the mechanical properties mentioned above, indicating that these properties are degraded with exposure time. 

The composites with CA showed less erosion after natural aging, probably due to better encapsulation and contact of the polymeric matrix with vegetable fiber, protecting it from weather conditions. Although the environmental conditions were the most aggressive (high UV index and highest rainfall) in the first six months, the resistance of these composites were better, with only the formation of small lines on the surface. In the following 6 months of exposure, it was observed the appearance of cracks caused by environment stress-cracking.

The degradation of properties in composites exposed to natural aging may be caused by the division of chains and the degradation that occurs in PP molecules as a result of photo-oxidation promoted by UV radiation. This trend is also evidenced by the presence of fissures on the surfaces of the exposed composites. Microscopic changes confirm the loss of tensile properties due to degradation [38,40,42]. Surfaces of the composites exposed to natural aging showed morphological properties indicating severe degradation, as evidenced by a fragile nature [41].

The fissures present on the surfaces of the composites may be due to the formation of environmental stress cracking, as explained above, temperature, rainfall variations during all seasons.

The first stage of PP degradation may be related to the breaking of a covalent chemical ligation that generates relative species and free radicals, which are responsible for propagation of the process. 

The initial generation of this of this species can be caused by agents such as high UV index, heat, biological attack, and chemical exposure, among others. These forms of initiation indicate the energy needed to break chemical bonds [53].

### 4.4. Optical Analysis: Luminescence and Gloss

The visual appearance is very important for the study of degradation in polymeric materials, these tend to lose brightness, beginning to yellow and lose its color (whitening), to present cracks and fissures on the surface, among other defects which reduce their useful life [54]. The exposure of the Polypropylene to the ultra-violet light causes the photo-oxidative degradation by radicular via, it is formed by breaking polymeric chains, in the tertiary carbon, it produces free radicals, leading to a decrease in the molecular weight and to the reduction in the mechanical properties [51].

**Figure 7:**
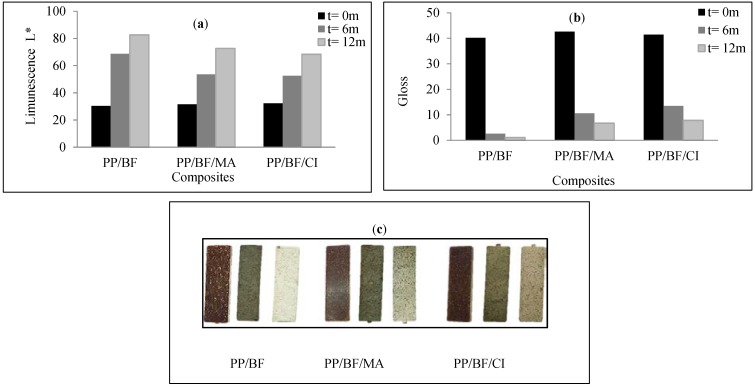
Physical properties: (**a**) Luminescence (**b**) gloss (**c**) photographs of the specimens.

Figure 7a,b, shows comparative graphics of the luminescence and gloss respectively, of the evaluated composites before and after exposure, and Figure 7c shows a better visualization of physical changes, from photographs of the specimens before (left figure) and after the exposure to natural aging (center and right figure for 6 and 12 months respectively). 

Figure 7a shows that all composites experienced an increase in luminescence or whitening (L index) along the 12 months of exposure, the composites without CA presented higher whitening, about 133% in the first six months and 180% at the end of 12 months of exposure that continuous increase, as shown in Figure 7c, the composites showed loss of color from brown to whitish (increase of L), this indicates that the climatic conditions solar radiation and rainfall produced the whitening in the composites and possible leaching of the extractives. According to Poletto et al. [55], the chemical composition of the extractives influences the color of the wood, and the removal of these components causes whitening. The composites without AC present higher whitening, with more fractures, along of exposure time; while the coupled composites showed lower L index, less intense whitish tone at the end of 12 months of exposure, the PP/BF/CI composites show slightly more stable surfaces than the PP/BF/MA composites, this behavior can also be verified in the micrographs, in general they are results coherent with the mechanical and thermal properties.

In Figure 7b the results referring to the gloss of the composites evaluated are shown, where it is observed that all composites experienced decrease of the gloss on the exposed surface, that was more intense in the first six months, by the formation of a rough surface, due that this period was characterized by extreme weather events, above mentioned (Table 2). The composites without CA presented higher loss, close to 100% at the end of 12 months of exposure. The PP/BF/MA composites showed a loss about 85%, and the PP/BF/CI composites showed the lower loss about 65%. These results indicate once more that the use of the CA provided higher interaction of the fiber with the polymeric matrix, where the PP matrix would be encapsulating fiber, protecting it from moisture and rain, slowing the hydrolytic degradation of the fiber [56,57,58]. In composites without CA, the weak of interaction facilitated the entry of moisture and water, allowing leaching of some substances with lower molecular weight (extractives and hemicellulose) and the hydrolytic degradation of the lignocellulosic fiber. 

### 4.5. Rheological Properties

**Figure 8:**
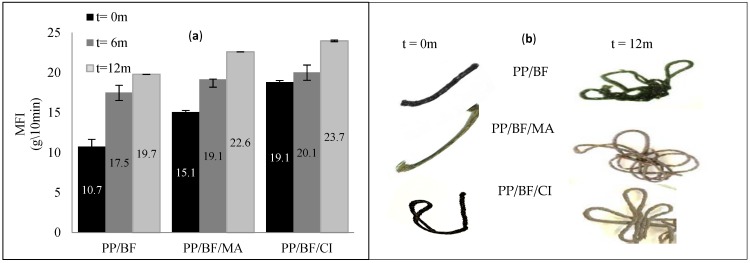
Results of melt flow index (MFI) of composites: (**a**) before and after 6 and 12 months natural aging; (**b**) pictures of samples tested by MFI before and after 12 months exposure.

Figure 8a shows the results of the melt flow index (MFI) expressed in grams per 10 minutes of the composites before and after (6 and 12 months) natural ageing, and the Figure 8b shows pictures of samples tested by MFI. It is noted that all exposed composites experienced an increase in MFI.

The influence of the incorporation of CA in the composites before exposure is observed, where it can act as a lubricant and/or plasticizer [22], increasing the fluidity of the composite, being superior the coupled with citric acid. A confirmation of this increase in MFI, is visualized in pictures taken of the samples tested by MFI, where the longest and thinnest spaghetti is of the PP/BF/CI composite.

The composites without CA presented lower MFI before exposure when compared with the composites with CA; that was also observed by Catto (2015) [59]. 

The initial MFI values (before natural aging) were different, it can be seen that the coupled composites showed higher MFI, probably due to the presence of CA, that besides being coupling agent, but it also acted as a lubricant [22], being higher in PP/BF/CI composites.

As shown in Figure 8, after six months natural ageing, all composites showed similar values of MFI, which means that the composite without CA had a significant increase (63%), in coupled composites the increase was lower of 18% and 7% for PP/BF/MA and PP/BF/CI, respectively. These results may indicate that the presence of CA provided stability in the composite, maintaining the function of promote the bonding between fiber and matrix, increasing resistance to environment stress cracking and to the temperature, throughout the natural aging process.

In the last six months, all composites presented increase in MFI, however, the percentage of increase was higher in composites with a CA of 17% and 19% for PP/BF/MA and PP/BF/CI composites respectively and in PP/BF composites the increase was 13%. This behavior may be due to the beginning of stress cracking that allowed the hydrolytic degradation of holocellulose [56] and photooxidation of PP [22], influenced by the polar functional groups of the CA.

These results after 12 months exposure can be observed in the pictures taken after the test (Figure 8b), where a larger amount of sample and smaller diameter after the test was verified, this confirms higher flow, thus a lower viscosity. 

These observations are consistent with the findings on the thermal properties mentioned above; the thermogravimetric analysis showed that the presence of CA in the PP/BF composites provided higher thermal stability.

The increase in MFI is related to the scission of polymer chains [34]. This behavior is a consequence of PP degradation mechanisms, which involves chain breakage due to the β scission of alkoxy radicals generated by PP self-oxidation [60]. The chain scission generally turns the low MFI commodity resins into high MFI resins [61]. This behavior in composites exposed under natural outdoor aging, has also been observed by other authors [60,62,63,64].

## 5. Conclusions

The PP is known as non-biodegradable polymer, but is sensitive to a series of thermal and photo-oxidative reactions, that affect the performance of the material occur weathering, a process in which the incidence of ultraviolet radiation and atmospheric oxygen interact, which was confirmed in this study after natural aging.

PP/BF composites are shown to be sensitive to natural aging; the degradation of their mechanical and morphological properties is dependent on the period of exposure and the climatic conditions.

The PP/BF composites were affected by degradation due to photodegradation in PP, and hydrolytic degradation of holocellulose favored by environment stress cracking and weather conditions.

This type of polymer matrix composite with plant fiber reinforcement needs protective agents to optimize its use in natural weathering.

Extreme climatic conditions experienced during weathering exposed, affected the performance of the material, the composites with CA had lower loss of their properties. 

The PP/BF composites with addition of CA showed better mechanical performance and stability to the natural ageing, and less affected surfaces than composite without CA.

The use of CA in PP/BF composites improve the thermal stability, according to the results of TGA analysis.

Finally, the CA of organic origin, (CI), showed similar results to the CA of synthetic origin (MA) with respect to the properties evaluated, so citric acid can act as a coupling agent in PP/bamboo fiber composites.

## Figures and Tables

**Table 1:** Formulations of PP/BF composites without CA and with CAs.

Composite	BF (wt %)	PP (wt %)	Coupling Agent (wt %)	
			MA	CI
PP/BF	30	70	-	-
PP/BF/MA	30	67	3	-
PP/BF/CI	30	67	-	3

**Table 2:** Data of the climatological variables analyzed for 6 and 12 months.

Climatological Variations Analyzed		6 Months	12 Months
		Exposure Period: 09/01/2016–09/07/2016	Exposure Period: 09/01/2016–09/01/2017
Temperature	Minimum average	19.1 °C	14.9 °C
	Registered minimum	11 °C	6.4 °C
	Maximum average	28.8	24.8 °C
	Maximum registered	36 °C	36 °C
	Medium daily variation	9.3 °C	9.6 °C
Rainfall	Average rainfall	6.6 m	3.6 mm
	Accumulated rainfall	943 mm	1224 mm
PH	Medium	3.7	5.7
	Minimum registered	3.0	4
	Maximum registered	7.9	7.9
UV Index	Medium	9.8	7.8
	Minimum registered	4	2
	Maximum registered	14	14

**Table 3:** Summary of the mechanical properties of the composites before and after natural aging.

Sample/Exposure Time (Months)		Elasticity Module (GPa)	Tensile Stress at Break (MPa)	Extension at Break (mm)	Impact Izod (kJ/m^2^)
PP/BF	0	1.40 ± 0.06 ^a^	12.45 ± 0.63 ^g^	0.99 ± 0.10 ^m^	11.00 ± 0.48 ^s^
	6	1.16 ± 0.03 ^b^	10.00 ± 0.29 ^h^	1.00 ± 0.10 ^n^	8.70 ± 0.79 ^t^
	12	0.85 ± 0.01 ^c^	7.80 ± 0.15 ^i^	0.80 ± 0.12 ^o^	7.8 ± 0.23 ^u^
PP/BF/MA	0	1.63 ± 0.10 ^d^	14.87 ± 0.55 ^j^	2.14 ± 0.31 ^p^	13.42 ± 0.92 ^v^
	6	1.45 ± 0.12 ^e^	13.63 ± 0.15 ^k^	1.24 ± 0.12 ^q^	11.23 ± 0.27 ^w^
	12	1.34 ± 0.07 ^f^	11.66 ± 0.50 ^l^	1.09 ± 0.13 ^r^	10.02 ± 0.71 ^x^
PP/BF/CI	0	1.58 ± 0.06 ^d^	15.17 ± 0.45 ^j^	2.27 ± 0.06 ^p^	12.89 ± 0.34 ^v^
	6	1.41 ± 0.08 ^e^	12.40 ± 0.55 ^k^	1.16 ± 0.07 ^q^	10.40 ± 0.46 ^w^
	12	1.32 ± 0.03 ^f^	11.93 ± 0.79 ^l^	1.08 ± 0.04 ^r^	9.71 ± 0.70 ^x^

^a–x^ Equal letters in the same column indicate that there are no significant differences between samples.

**Table 4:** Results of the thermal properties (TGA and DTG) of composites evaluated.

Sample	Time (Month)	TGA		DTG			
		*T* (°C) 10% wt	Ash (%)	1st *T*_p_ (°C)	2nd *T*_p_ (°C)	3rd *T*_p_ (°C)	4th *T*_p_ (°C)
PP/BF	0	310	7.5	84	259	330	475
	12	316	2.3	86	265	333	457
PP/BF/MA	0	320	6.1	90	286	348	464
	12	319	4.8	92	299	*357*	467
PP/BF/CI	0	333	1	96	295	357	451
	12	328	1.6	98	289	357	461

*T*_p_: peak temperature from DTG curve of each decomposition stage.

**Table 5:** Results of the thermal properties and crystallinity index obtained by DSC.

Sample	Time (Month)	2nd Heating		
		*T*_m_ (°C)	∆*H*_m_(J/g)	*X*c (%)
PP/BF	0	165	74.1	50.89
	12	166	63.2	43.41
PP/BF/MA	0	166	58.7	40.32
	12	165	70.6	48.49
PP/BF/CI	0	166	60.4	43.34
	12	164	64.8	46.50
